# Treating clients with Asperger’s syndrome and autism

**DOI:** 10.1186/1753-2000-7-32

**Published:** 2013-09-11

**Authors:** Alisa G Woods, Esmaeil Mahdavi, Jeanne P Ryan

**Affiliations:** 1 Chemistry and Biomolecular Science, Clarkson University, 8 Clarkson Avenue, Potsdam, NY 13699-5810, USA; 2 Neuropsychology Clinic and Psychoeducation Services, SUNY Plattsburgh, Plattsburgh, NY 12901, USA; 3 Mental Health Counseling Program, College of Education and Human Development, University of Massachusetts, Boston, 100 Morrissey Blvd, Boston, MA 02125, USA

**Keywords:** Asperger, Autism, Counseling, ASD, Autistic, High-functioning-autism

## Abstract

Asperger’s syndrome (AS) is a form of autism spectrum disorder (ASD) affecting many individuals today. Although neurobiological correlates for AS have been identified, like many ASDs, AS is not completely understood. AS as a distinct disorder is also not universally accepted and in the DSM-5 AS is not considered a separate nosological entity. In contrast to some other ASDs, individuals with AS are commonly characterized by having standard or higher than average intelligence, yet difficulties in social skills and communication can present challenges for these individuals in everyday functioning. Counseling a person with AS or autism presents a unique challenge for the mental health care provider. We have compiled this review consisting of some recent ideas regarding counseling the client with AS with the goal of providing some clinical insights and practical clues. Although the focus of the present paper is largely on AS, many of these strategies could also apply to individuals with high-functioning autism (HFA).

## Introduction

Autism spectrum disorders (ASDs), including Asperger’s syndrome (AS), have been by the presence of a “triad” of impairments: 1) social deficits, 2) repetitive/stereotypical behaviors and interests and 3) communication difficulties [1]. Additional ASDs have in the past included Autism and pervasive developmental disorder not otherwise specified (PDD-NOS) [2]. AS was originally described by Asperger in 1944 [3] but was not included in the DSM-IV until 1994 [4]. In May 2013 the DSM-5 was introduced, subsuming both AS and PDD-NOS under the umbrella term ASD. This will now be used to classify all of the discrete autistic disorders previously classified separately on the DSM-IV. Clinical aspects are divided into a dyad of impairments: 1) persistent deficits in social communication issues and 2) restricted, repetitive behavior, interests and activities [5]. The extent to which the DSM-IV and DSM-5 diagnoses will overlap is currently uncertain and concerns have been expressed that some individuals previously diagnosed with AS will not qualify for ASD diagnosis on DSM-5 and that appropriate services will not be received by everyone who requires them [6].

At least one study indicated that the draft DSM-5 criteria were less sensitive in diagnosing individuals previously diagnosed with AS with an ASD [7]. It should be noted that the WHO ICD-10 system retains the classification of AS and is not due to be revised until the introduction of ICD-11 in 2015. Lack of diagnosis and treatment of AS is already a concern. Since individuals with AS are of average intelligence or above, they may fall into a therapeutic “no man’s land” and be considered too high-functioning for disability services [8,9]. In contrast, other studies have suggested that the impact of the revisions may be to reduce false positive diagnoses of ASDs [10] or that most children with DSM-IV ASD diagnoses will remain diagnosed under DSM-5 criteria [11].

## Prevalence

Recent estimates indicate that 1 in 88 US children have an ASD, including AS. ASDs in general occur more frequently in boys than girls; one in 54 boys and one in 252 girls are affected [12] and this gender bias is also true of AS. A United States government survey (Centers for Disease Control and Prevention; CDC) of parents indicates that the prevalence may even be as high as 1 in 50 [13]. The overall prevalence of AS is not completely clear, however, it has been estimated that 2–7 out of every 1,000 children have AS, and AS is 2–4 times more common in boys than girls [14,15]. ASD incidence in general appears to be on the rise, although the contribution of a true increase versus improvements in awareness and diagnosis is not currently known. It is believed by many that improved detection alone does not account for increases in ASD prevalence [16].

## Distinction from other ASDs

AS has been largely distinguished from other ASDs based on a lack of delay in cognitive development (other than social), and a lack of delay in language development [2]. Neither a lack of nor a delay in cognitive or language development was a DSM-IV or is an ICD-10 exclusion criterion for an autism diagnosis. For some this created confusion as it did not allow clear demarcation of AS, while for others it marked the need for additional discriminators such as motor function and neuropsychological profiling. Individuals with AS are said to have a higher probability of presenting with motor difficulties (clumsiness), and some groups, though not all, have also reported that they are more likely to show a non-verbal learning disability profile in contrast to those with an HFA diagnosis of similar overall cognitive ability [17]. AS is therefore often diagnosed later than other ASDs, (after age three or so) [18]. Distinguishing AS from high-functioning autism (HFA) or PDD-NOS in individuals without cognitive problems can be difficult [19], and it is this lack of distinction that has led to the elimination of these terms in DSM-5. There is a continuing controversy regarding whether or not these are in fact distinct disorders [20].

Symptomatically, people with AS have been described as having poorer motor skills and better language than people with HFA [21]. A study of neuromotor behavior has further supported behavioral distinctions between AS and HFA, based on gait patterns [22]. A neuroimaging study using magnetic resonance imaging indicated that children with AS do seem to comprise a distinct ASD subgroup, based primarily on grey matter volumes. In comparison to children with AS, children with HFA had significantly smaller grey matter volume in subcortical, posterior cingulate and precuneus regions of the brain. Children with AS had less grey matter in mainly bilateral caudate and left thalamus versus non-ASD controls [23]. In addition, brain white matter volumes in several anatomical locations are distinct in children with AS from both children with HFA and controls, possibly indicating different patterns of brain connectivity in people with AS [24]. This neuroanatomical evidence was interpreted as supporting that AS is a distinct disorder. Other neuroimaging studies have further supported neuroanatomical differences in AS versus HFA [25,26]. A recent article utilizing EEG examined patients aged 2–12, diagnosed with either ASD (n = 430) or AS (n = 26). Neurotypical controls were also studied (n = 554). Although the individuals with AS were neurophysiologically more similar to individuals with ASD than controls, they also appeared to represent a population that could be distinguished from subjects with ASD, based on EEG patterns [27]. In contrast, a meta-analysis of gray matter abnormalities in ASD (24 data sets) failed to identify differences in overall or regional gray matter volumes between individuals with autism versus AS [28]. A diagnostic study has suggested that distinctions among subtypes of ASDs, specifically autistic disorder, PDD-NOS and AS, were not reliable across sites using standardized diagnostic tools [29].

As mentioned, the DSM-5 does not distinguish between AS and autism, while the ICD-10 does. Although the term AS is used throughout this paper and the sources cited largely also assume that AS is a distinct diagnosis, we also acknowledge the possibility that autism and AS are the same condition. Equally, it may prove to be the case that there are a range of distinct ASD conditions that can be discriminated. This issue is the subject of continuing research. Over the period from 2000 to 2012 studies published on AS using ICD or DSM diagnosed populations were based on broadly comparable criteria, while earlier work varied in the AS criteria employed.

It should be noted that the literature on treatment differs in the diagnostic criteria used and clinical groups studied. The therapeutic strategies reviewed may apply to individuals with AS or HFA regardless of the diagnostic definition used, but their applicability needs to be tested where only work with AS or autism has been reported.

## Social difficulties

Individuals with AS may have conventional vocabularies but they typically have problems with social communication, particularly conversation, non-verbal cues and reciprocal interaction [30-33]. They have been said to have difficulties in empathy and theory-of-mind (the ability to distinguish between one’s own mental state and that of others) [34,35]. Individuals with AS may have trouble processing social meaning due to having these challenges. According to one study comparing 15 adults diagnosed with AS versus 15 non-ASD control subjects, adults with AS experience social cognition deficits, including the decreased ability to implicitly encode and integrate contextual information to access social meaning. However, if abstract rules specific to a social situation are presented, or if social information is given in an explicit manner, adults with AS can perform in a social situation more appropriately [36].

Social awkwardness can be a vicious cycle for people with AS, since they lack social skills which in turn can prevent them from forming relationships with other people and thus hinders the development of these skills [37]. The social isolation and difficulties caused by AS frequently result in reduced quality of life, and may increase the incidence of depression and anxiety [38]. Social anxiety in individuals with AS has been said to be distinct from conventional social anxiety, since people with AS are confused about social rules whereas non-AS individuals with social anxiety may be ruminating about social rejection [35]. Depression may be directly related to social problems in people with AS [39]. Anxiety is also common [40], likely based on social difficulties and sensory overload. Therapists are often in the position of providing guidance regarding social interactions when counseling people with AS [35]. Therefore, social skill development and understanding of social interactions may take an important role in therapy with individuals with AS.

Individuals with AS may have a tendency to persistently focus on specific topics in conversational speech, or conversation may take on an instructional, sometimes patronizing tone or may be overly formal or pedantic [32,41]. Naturally these patterns of conversation are important for counselors and therapists to be aware of when working with clients with AS. Counselors may want to remind themselves that these are common characteristics found in individuals with AS. It may be important in addition for the therapist to note that people with AS do not always understand the effect their behavior has on others [34]. Pedantic speech may indicate that the client is anxious or uncomfortable and therefore reverting to a habitual pattern of speaking. People with AS may experience social interactions as generally anxiety-provoking [37,42], which makes formation of a therapeutic alliance in clients with AS particularly challenging. Predictability of a therapeutic environment can help clients with AS to feel safe, therefore therapists might want to consider any ways in which the therapeutic environment or the routine of therapy could be made to be consistent [42]. Sincerity on the part of the therapist is also crucial, since these individuals have been proposed to have little tolerance for falsity [37], perhaps because they do not understand the social utility of insincerity in some situations. Therefore, it may be advisable to put concerns into simple, direct terms, even if it means telling the individual with AS directly that that person is engaging in an undesirable behavior.

Social difficulties are common in AS, and consequently many research studies have focused on interventions directed at improving social interaction. One study described the development of socio-dramatic affective-relational intervention’ (SDARI), a six-week program. SDARI is a group, manualized intervention that utilizes 1) a performance-based social skills curriculum consisting of improvisation games and dramatic training specifically developed for people with AS 2), a focus on dyadic relationships between child-child and child-staff for reinforcing social interactions, and 3) additional age-specific motivators (for example, video games). Nine participants age 11–17 with ASD participated in the study and were compared to eight age-matched individuals with AS who served as a control group. The control group did not participate at the time of the study but these individuals may or may not have participated at a different time. Outcome was measured through parent and child reports of social functioning. These were collected at three week intervals, starting three weeks prior to the training and continuing until six weeks after completion. The SDARI program participants experienced improved ability to detect emotion in adult voices from baseline relative to controls and in assertion, and the gains persisted up to six weeks following the treatment [43].

In addition to group training, individual social skills training using a cognitive-behavioral framework has also proven effective for improving social behaviors in individuals with high-functioning ASDs, including AS [44]. The use of video modeling for enhancing conversation skills has been supported in a case study of a boy with AS [45]. A further, two-subject study in college students reported a significant positive change in eye contact, facial expression, and conversational turn-taking for one participant and in eye contact and turn-taking for the other [46]. Social-behavioral learning strategy training (Stop-Observe-Deliberate-Act; SODA) has also been used to improve social interaction skills in four students with AS [47]. In this study, all four participants increased the time they spent in cooperative learning activities, in playing sports and in visiting peers at lunchtime.

A further study compared LEGO therapy (n = 16), a Social Use of Language Program (SULP) (n = 15) and no therapy control group (n = 16) in children age 6–11 with HFA or AS. LEGO therapy, which involved building models using LEGO materials in pairs or small groups, was found to produce greater improvement than either SULP or no therapy on autism specific social interaction scores (using the Gilliam Autism Rating Scale). Maladaptive behavior significantly decreased to a greater extent in the LEGO and SULP groups compared to the no therapy controls [48].

Therapists may consider incorporating components from any of these four approaches (SDARI, video modeling, SODA, LEGO therapy) in their own work with individuals with AS focusing on social skills and may refer to the cited work to better understand these approaches. We should note however, that the literature cited is most substantive for the LEGO approach and that the other studies were conducted on small, unreplicated samples. Therefore, it may be advisable to monitor the effects of the other interventions carefully.

Group training, if available, may be valuable because it provides opportunities for structured social interaction. Therapists might encourage clients with AS to utilize their special skills to increase opportunities for socialization. One case study examined increases in social acceptance of a boy who engaged in collaborative computer work in school with his peers. The authors of this study reported that the experience improved the boy’s ability to interact with peers and seemed increase his social profile with classmates in general [49]. Unfortunately, like many studies examining social skills training in AS, this report is a case study, therefore although the approach may appear promising, more rigorous evaluation is needed. The case does illustrate the concept, however, of using special skills (computer work) to engage in social interactions with others.

A review of studies on the effects of social skills training on children and adolescents with ASDs in general, found that out of ten social skills training studies, seven reported positive effects of treatment. Unfortunately, several problems in interpreting across studies were identified, including lack of a universal social skills definition, differences in levels of intensity or duration of social skills training, differences in services provided in the clinic or classroom. Study sizes were small and tended not to control for maturation and time over treatment. Raters also tended to be unblinded. It was concluded that social skills training is widely used, but there is a dearth of strong empirical evidence supporting the use of social skills training in AS and HFA. The authors recommended that there is further research to document the specific deficits in youth with AS and HFA, that the effectiveness of social skills training should be further clarified through randomized clinical trials, and that training programs should include a focus on generalization of training effects outside of the treatment setting [50]. Williams White et al. conducted a separate review of all published studies of group social skills interventions for individuals with ASDs between 1985 and 2006. This review also concluded that studies of social skills training in ASDs currently lack empirical rigor. Based on 14 studies, the authors proposed the following goals for social skills training in ASDs: 1) increased social motivation, 2) increase social initiation, 3) improvement in appropriate social responding, 4) reduction of interfering behaviors, and 5) promotion of skill generalization [51].

A Cochrane systematic review examined sixty- six studies of social skills training in ASDs published in peer-reviewed journals between 2001 and July 2008. The studies reviewer reported on 513 participants aged 6 y-21 y. It was concluded that a number of treatments targeting social deficits in autism were supported by the studies reviewed [52]. From 2004, group designs began to be reported and have greatly strengthened the evidence-base for social skills training. The review did not focus on social skills training in AS per se, however the use of social skills groups and video modeling treatments for autism in general were supported by this literature [52]. Several methods for addressing social skills training have been identified and evaluated and have empirical support for their possible efficacy in working with AS clients.

## Restricted interests

Individuals with AS may have extremely restricted interests. In some cases (though not always), restricted interests can manifest as “special interests” ie., hobbies. One clinician has suggested that the special interests of AS clients can be utilized in counseling, for example, a client with a special interest in Star Trek was once asked “what would Mr. Spock do in a situation like this?” [8]. Asking the client about interests may also serve as an initial means for developing an alliance and increasing the comfort of the client with the therapy setting [37,42]. One client with AS discussed the composition of meteors at length when asked about them by a therapist and seemed pleased to be consulted about his expertise on the subject. This initial half hour of discussion preceded a final 20 minutes of greater self-disclosure and seemingly more comfort with the therapy setting.

## Linguistics

Individuals with AS typically speak fluently but have problems engaging in reciprocal conversation. Their conversational tone may be unusual and/or pedantic. Clients with AS may have difficulty understanding metaphors [53]. For example, in response to the phrase “have your cake and eat it too” a client with AS stated “I don’t understand what that means, except that it has something to do with cake.” It has been proposed that difficulties with metaphors may be attributed to difficulties in processing language in the right hemisphere [53]. The right hemisphere is more responsible for distant and figurative meaning, as opposed to the more literal meanings of words processed by the left hemisphere [54]. Irony and implied meaning can also be confusing to a person with AS [55]. These traits can also become apparent within the counseling setting and the counselor may therefore want to speak to a client with AS using very literal language and avoid metaphors or figures of speech. In general, it may be helpful to take a direct, instructional approach versus a more indirect approach that encourages clients to uncover solutions to their own problems [35]. This approach may be counter-intuitive to many therapists.

## Emotionality

Emotion and emotional reactions may be difficult for individuals with AS to process and discuss. This can include the expression of emotions, understanding the emotions of others, and emotion regulation. Individuals with AS may not have diminished emotionality, however it may be more accurate to say that individuals with AS have greater difficulties in understanding and compartmentalizing their own emotions or in conveying these to others.

Neuroimaging studies have indeed shown that in people with AS the amygdala and hippocampus—interconnected brain regions essential to processing emotions and emotional memories—appear to have differences in their neuronal and lipid composition relative to non-AS controls [56]. It may be that emotions overwhelm people with AS, increasing their inability to manage them. Depression and anxiety higher in people with AS than in the general population [57]. Depression and anxiety may be further exacerbated by the fact that people with AS are often victims of bullying and abuse, likely due to their social awkwardness [8]. Therapists treating individuals with AS may therefore consider screening for mood disorders as part of the therapy initiation process. One study has suggested that individuals with autism in general tend to express anxiety through changes in their behavior rather than verbally. Therapists may therefore note that depression and anxiety will not always be verbalized by the client, but may become apparent through behavioral changes [58].

A randomized controlled trial examining a six-week cognitive behavioral intervention demonstrated decreased anger episodes in 24 children with AS relative to 21 children with AS who did not undergo the intervention [59]. Single subject case reports have additionally suggested that cognitive behavioral therapy (CBT) can be successful at treating anxiety, depression and social problems in clients with AS [60,61]. CBT, specifically in a group setting, was also found to be helpful for adults with AS or mood disorders [57]. The authors of a three case-study report suggested that group therapy could be more effective than individual therapy for people with AS because it provides often isolated individuals with AS with support and also opportunities for social learning, which is greatly needed by individuals with AS. The authors of this report also noted that the three participants appreciated the structure and scientific nature of CBT and their ability to chart their progress [57]. In addition to the use of CBT for managing one’s own emotions, interactive media have also been employed to teach adults with AS to effectively learn to recognize complex emotions in others [62]. Based on these studies, emotional problems can be effectively treated in individuals with AS. There is supporting evidence for a CBT approach either working individually or in a group format. This conclusion is supported by several studies. Therapists may consider using CBT in their treatment of individuals with AS.

We would also like to note that in our experience clients with AS may appreciate a technique such as CBT if it (and the underlying science) is explained to them. They are not very appreciative if they sense that a therapist is “using a technique” without their knowledge and may consider this insulting to their intelligence. One client encountered a therapist who was using the non-directive technique of paraphrasing the client’s own words back to him, to try to show empathic understanding. The client stated “why do you keep saying what I say in different words?” The client with AS concluded that the therapist was trying to underestimate his intelligence and refused to see that therapist again. In general, it is best to express emotions directly with these individuals, as they are unlikely to “intuit” what is unspoken.

## Sensory and motor problems

People with AS, like other individuals with ASDs, are often susceptible to sensory overload [37]. A therapists office for example may be too bright, too loud, etc., for the individual with AS. It may be valuable to ask someone with AS whether there is something in the environment that is overwhelming for that person. This may also be appreciated by the client as an empathic gesture that shows the therapist is aware of difficulties specific to AS [37]. Conversely, people with AS may be hyposensitive, for example to pain or may have other sensory impairments [37].

In addition to the above-mentioned symptoms, people with AS also often have some degree of motor clumsiness [18]. Cognitive strategies, known as Cognitive Orientation for (daily) Occupational Performance (CO-OP) have also been utilized for improving motor performance in individuals with AS [63]. This intervention is a child-focused goal-directed technique used by occupational therapists to help motor-impaired individuals reach functional goals [64]. If occupational therapy is available, this may be something for the client with AS to consider. Structured physical activities, such as exercise or dance classes may be another option to consider for improvement of motor control. An exercise study in 15 children with ASD (age 5–16) compared to a no-exercise ASD control group (n = 15) founds that teaching martial arts techniques to children with ASD four times per week for 14 weeks consistently decreased their stereotypic behaviors [65].

## Conclusions

Counseling individuals with AS and HFA has some distinctions compared to other counseling populations. A great emphasis will often be placed on social interactions and understanding them. We have presented some studied interventions for enhancing social skills however counselors may also consider an instructional approach, in which they explain social interactions and social rules. This is particularly recommended if group therapy or group instruction is not available or if they client is uncomfortable with a group setting. It should be remembered that although clients with AS and HFA may not express emotion in a conventional matter, they are not devoid of emotion. In contrast, they may have emotional difficulties, including problems managing anger, or mood disorders. Emotional problems and mood disorders are important to monitor for in these clients. AS and HFA clients can be overwhelmed by their own emotions, as they often are by sensory input, and their outward behavior does not always reflect their inner state. The therapeutic setting can provide a safe, consistent, controlled space where they can begin to unravel the mysteries of their own emotions, as well as begin to understand the emotions of others through the therapists’ gentle explanation. Empathy and genuineness are important characteristics when counseling all clients, but perhaps even more so with clients with AS, who tend to be confused by insincerity. AS clients do not usually respond to implied meanings and appreciate literal and concrete explanations.

Counseling clients with AS and HFA may seem a daunting prospect at the outset, however therapy with these individuals can be very rewarding. Some resources have been developed that may be useful for clinicians treating individuals with AS and autism. For example, Gaus has adapted traditional CBT methods in her work with adults with AS [66], and Jacobsen has written a book describing psychotherapy methods with individuals with AS [67].

Overall, in the field of AS and HFA psychotherapeutic treatment much further work is required, conforming to comparative evaluation research criteria. Therapists with insight into ASD problems need to provide adapted, tailor-made, and insightful therapies to best address the specific needs of this group. More rigorous scientific research is required to be able to document better what kinds of therapy can be advocated, and what kind of gains and improvement might be expected.

## Abbreviations

ASD: Autism spectrum disorder; AS: Asperger’s syndrome; CBT: Cognitive behavioral therapy; CO-OP: Cognitive orientation for (daily) occupational performance; DSM: Diagnostic and statistical manual; HFA: High-functioning autism; PDD-NOS: Pervasive developmental disorder not otherwise specified; SDARI: Socio-dramatic affective-relational intervention; SODA: Stop-observe-deliberate-act.

## Competing interests

The authors declare that they have no competing interests.

## Authors’ contributions

AGW was responsible for writing the manuscript. EM and JR commented on the written drafts of the manuscript. All authors read and approved the final manuscript.
